# The Role of Lipid Metabolism in T Lymphocyte Differentiation and Survival

**DOI:** 10.3389/fimmu.2017.01949

**Published:** 2018-01-12

**Authors:** Duncan Howie, Annemieke Ten Bokum, Andra Stefania Necula, Stephen Paul Cobbold, Herman Waldmann

**Affiliations:** ^1^Sir William Dunn School of Pathology, University of Oxford, Oxford, United Kingdom

**Keywords:** T cell, fatty acid, metabolism, regulatory T cell, tolerance, lipotoxicity, Th17, cholesterol

## Abstract

The differentiation and effector functions of both the innate and adaptive immune system are inextricably linked to cellular metabolism. The features of metabolism which affect both arms of the immune system include metabolic substrate availability, expression of enzymes, transport proteins, and transcription factors which control catabolism of these substrates, and the ability to perform anabolic metabolism. The control of lipid metabolism is central to the appropriate differentiation and functions of T lymphocytes, and ultimately to the maintenance of immune tolerance. This review will focus on the role of fatty acid (FA) metabolism in T cell differentiation, effector function, and survival. FAs are important sources of cellular energy, stored as triglycerides. They are also used as precursors to produce complex lipids such as cholesterol and membrane phospholipids. FA residues also become incorporated into hormones and signaling moieties. FAs signal *via* nuclear receptors and their channeling, between storage as triacyl glycerides or oxidation as fuel, may play a role in survival or death of the cell. In recent years, progress in the field of immunometabolism has highlighted diverse roles for FA metabolism in CD4 and CD8 T cell differentiation and function. This review will firstly describe the sensing and modulation of the environmental FAs and lipid intracellular signaling and will then explore the key role of lipid metabolism in regulating the balance between potentially damaging pro-inflammatory and anti-inflammatory regulatory responses. Finally the complex role of extracellular FAs in determining cell survival will be discussed.

## Introduction

### How Are Dietary Lipids Sensed by Cells, How Do They Signal?

#### Free Fatty Acids (FFAs)

**Figure 1:**
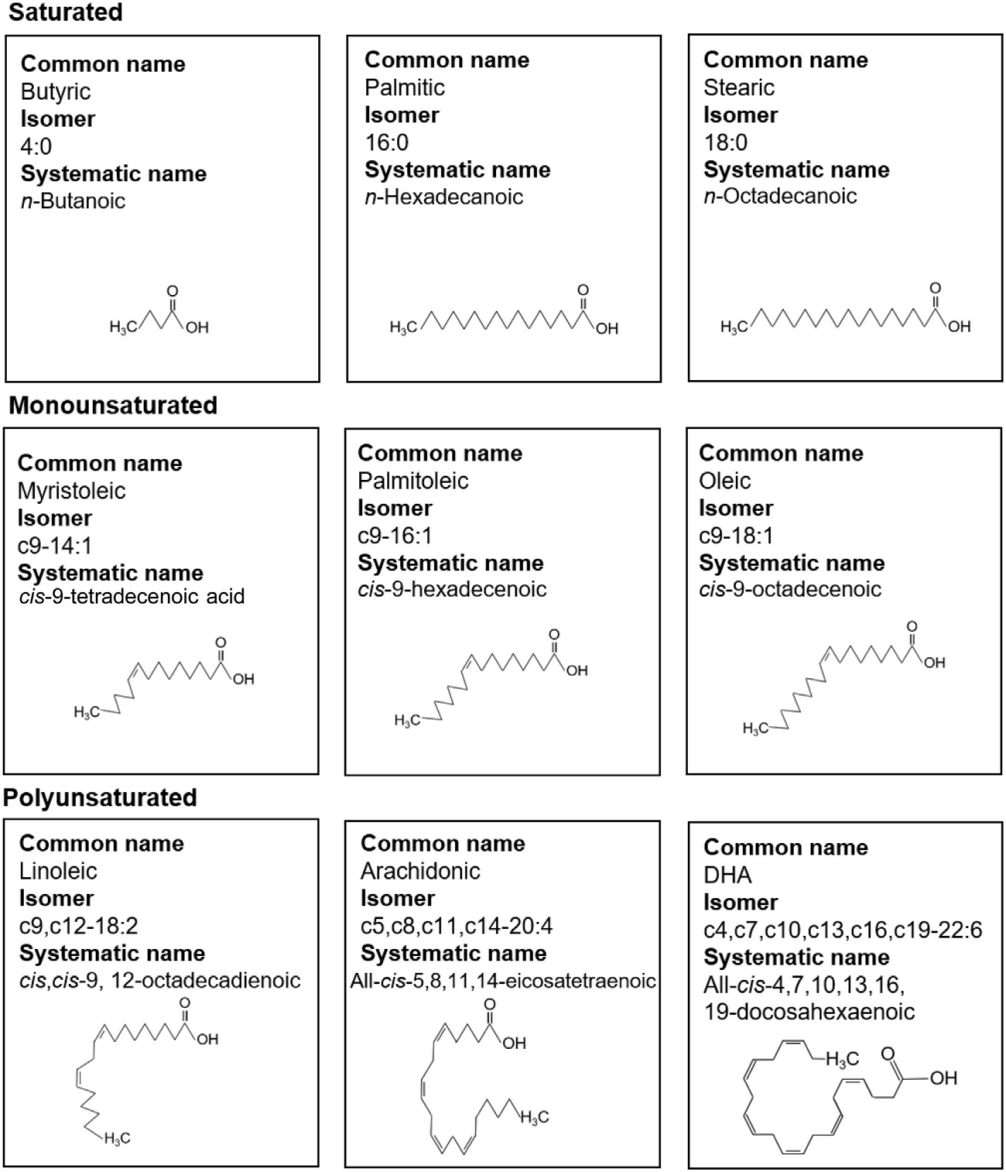
Fatty acid (FA) nomenclature. Common names, isomer formulas, systematic names, and structure of common saturated, monounsaturated, and polyunsaturated FAs.

Free fatty acids are defined as those not bound to albumin or esterified into larger molecules such as triglycerides (TGs) or phospholipids. FFAs have a simple structure of an aliphatic chain of varying length linked to a carboxyl group (Figure 1). Fatty acids (FAs) are classified according to their length in carbon atoms, their degree of saturation and whether their double bonds are in *cis* or *trans* orientation. For example, oleic acid, an 18 carbon unsaturated long-chain fatty acid (LCFA), can be abbreviated c9-18:1 indicating it has one *cis* double bond at the ninth carbon atom counting from the carboxyl terminal. FAs with 2–6 carbon atoms are termed short-chain fatty acids (SCFAs), 6–12 as medium-chain fatty acids (MCFAs), 14–18 as LCFAs, and over 20 as very long-chain fatty acids (VLCFAs). Essential FAs (i.e., those which the human body cannot produce) are predominantly diet derived. SCFAs such as propionic acid (C3:0) and butanoic acid (C4:0) are produced by bacteria residing in the gut lumen as a result of fermentation of fiber or dietary carbohydrate (1–5). They have a role in Treg homeostasis as will be discussed later.

#### Signalling

CD4 and CD8 T cell subsets are heavily dependent on, and influenced by, extra and intracellular FA content for their functions. These cells discriminate between both quantity and quality of FAs. Depending on these parameters, cell fate decisions are made resulting in changes to memory, subset differentiation, pathogenicity, and survival. Before these FA-influenced cellular decisions are made the cells have to recognize FAs, transfer them from the extra- to intracellular environments, signal to nuclear receptors, and convert the FAs into storage TGs or use them as fuel. The mechanisms of FA transport and signaling are diverse. There are numerous binding proteins and receptors for FAs that enable them to remain soluble in the extracellular environment, signal at the plasma membrane, be transported within cells and enable promotion of transcription factor activity. These will be discussed in turn.

#### Extracellular Transport

The human body requires approximately 0.3 mol FA to be transported from adipose tissue to fat-consuming tissues every 24 h (6). This requires approximately 0.3 mM FA concentration in the blood plasma (6). However, FAs have a much lower solubility than this in aqueous solution (7). To enable the concentration in plasma to be elevated to the required level FAs are transported around the body *via* lymphatics and blood in two ways. First, they are made soluble as TGs associated with chylomicrons and very low-density lipoproteins and second, as non-esterified FAs non-covalently bound to albumin. Albumin is an abundant 585 amino acid globular protein (8) containing 17 disulfide bridges (9), imparting great stability to the molecule with a half-life of around 20 days (9). Around 40 g is produced by the liver per day, and one-third to two-thirds of total albumin is in the interstitial compartment (10). Albumin has around seven binding sites for FAs of moderate to high affinity (6). Albumin is the major fatty acid-binding protein (FABP) in blood and interstitial fluid. Binding of FAs to albumin increases their concentration by several orders of magnitude.

#### Plasma-Membrane FA Receptors

Fatty acids have pleiotropic effects on T cells that depend on the mode of T cell activation, length of the FA, and degree of saturation in addition to the degree of metabolic substrate availability in the cell’s environment. In order for extracellular FAs to exert signaling or metabolic consequences on cells they first need to be recognized and/or taken up by the cell. T cell-surface receptors for FAs include G protein-coupled receptors (GPCRs), CD36, fatty acid-binding protein TM (FABP_TM_), and members of the fatty acid transport protein (FATP) family.

#### G Protein-Coupled Receptors (GPCRs)

Five cell-surface GPRs specific for FAs have been described; GPR 40, 41, 43, 84, and 120. They all have different affinities for FAs of different lengths. GPR41 and 43 have specificity for SCFAs, GPR84 for MCFAs, and GPR40 and GPR120 for LCFAs. However, of these only GPR84, the medium-chain FA receptor has been shown to be expressed by CD4 and CD8 T cells (11). GPR43 has high affinity for SCFAs and has been reported to be expressed by colonic Treg (cTreg) (4). There is some uncertainty about the degree of expression of the SCFA-binding GPRs GPR41 and 43 on colonic T cells (12–14). Expression of SCFA-binding GPRs may be context- or T cell subset dependent.

#### CD36

**Figure 2:**
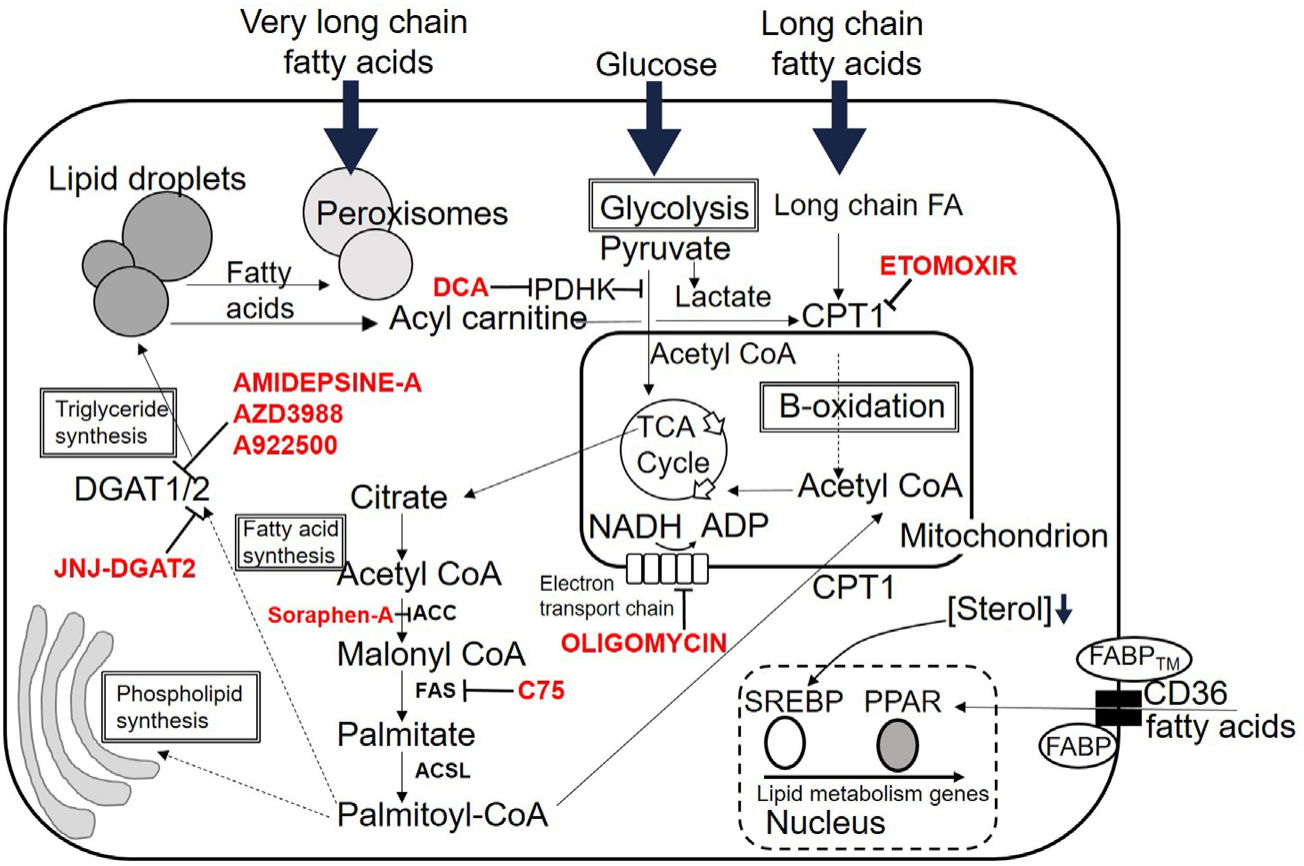
Fates of lipid within the cell. Overview of possible intracellular destinations of fatty acids. Commonly used inhibitor drugs for key pathways are shown in red. Dotted lines indicate multiple intermediate steps not shown due to space. See text for details. Abbreviations are listed at the start of the review.

Fatty acids may enter T cells through two basic processes. First, there is some evidence that they may enter the cell by passive diffusion, as T cells incorporate FAs into their membranes from their environment (15, 16). FA uptake at the plasma membrane is mostly controlled by membrane transport proteins such as CD36, plasma membrane-associated FABP, and FATPs. CD36 also known as fatty acid translocase is an integral plasma-membrane glycoprotein found on the surface of many cell types. It imports LCFAs inside cells and is a member of the class B scavenger receptor family of cell-surface proteins. CD36 binds many ligands in addition to FAs including oxidized phospholipids (17), oxidized low-density lipoprotein (LDL) (18, 19), native lipoproteins (20), and collagen (21). It has a hairpin membrane topology with two heavily glycosylated transmembrane regions (22). CD36 binds through its extracellular portion to the plasma-membrane FABP, FABP_TM_ and through its cytoplasmic portion to cytoplasmic FABP. The concerted action of this complex of three transport/chaperone proteins is thought to facilitate the diffusion and stabilization of FAs into T cells (Figure 2) (22).

#### Fatty Acid-Binding Proteins

**Figure 3:**
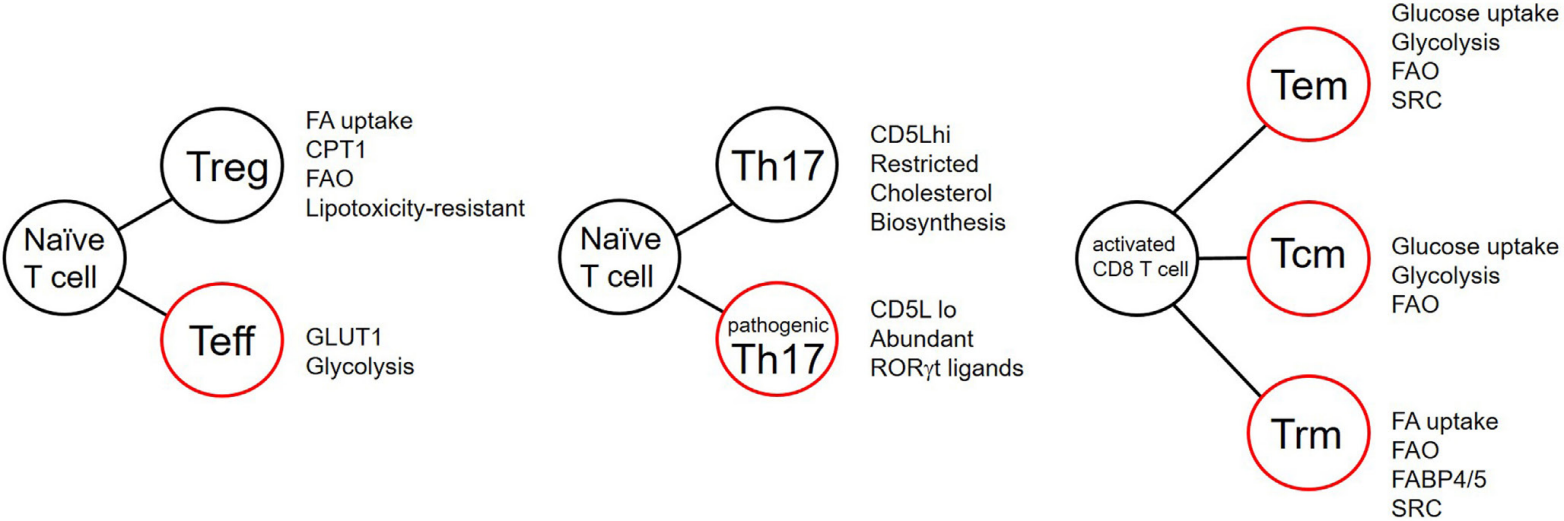
T cell differentiation and the effects of lipid metabolism. Summary of reported metabolic differences between T cell subsets during differentiation. Listed attributes indicate equal or increased features. FA, fatty acid; CPT1, carnitine palmitate transferase; FAO, fatty acid oxidation; GLUT1, glucose transporter 1; SRC, spare respiratory capacity; FABP, fatty acid-binding protein. Cells outlined in red indicate potentially inflammatory subsets.

Once inside the cell FAs are bound by FABPs to increase their aqueous solubility in the cytoplasm and to chaperone them to the correct cellular locations. FABPs comprise a family of nine proteins, most abundantly expressed in tissues involved in lipid metabolism. They can be divided into two groups, those associated with the plasma membrane (FABP_TM_) and cytoplasmic FABPs (FABP_c_). Each FABP has a different ligand specificity. For example, FABP1 and 5 bind to saturated, monounsaturated, and polyunsaturated FAs with no preference for any of these while FABP3 binds n6PUFAs such as arachidonic acid (23). FABPs have been proposed to coordinate FA uptake, stabilization, transport, and synthesis of FAs (24, 25). They may act as gatekeepers to the nucleus, regulating entry of FAs which signal *via* peroxisome proliferator-activated receptors (PPARs), discussed below. FABP4 and 5 are upregulated in a subpopulation of resident CD8 memory cells (Figure 3) and are critical for memory function (26), discussed later.

#### Fatty Acid Transport Proteins

The FATP family, also known as the solute carrier family 27, are a group of six transmembrane transporters with a heterogeneous tissue and cell distribution. These proteins transport VLCFAs into the cell where they are simultaneously converted into very long-chain acyl-CoA esters. This esterification results in “metabolic trapping” of the FA within the cell, a process also known as “vectorial acylation” (27). A similar process occurs with the hexokinase-mediated phosphorylation of glucose. Although a certain level of redundancy exists with FATP and CD36 and FABP_TM_, single-nucleotide polymorphisms (SNPs) in FATPs may predispose carriers to elevated risk of metabolic disease. For example, SNPs in FATP1 are associated with increased plasma TG levels (28, 29).

## FA-Signaling Receptors

### Peroxisome Proliferator-Activated Receptors

Once in the cell, FAs have multiple fates. In addition to fueling mitochondrial respiration, they also signal *via* nuclear receptors to alter transcription of genes important for lipid homeostasis. PPARs are a subset of the family of nuclear hormone receptors, transcription factors which are activated by lipophilic molecules (30) and control genes mostly involved with lipid metabolism. The PPARs consist of an N-terminal ligand-independent activation domain, a conserved DNA-binding domain, a C-terminal ligand-binding domain, and a C-terminal ligand-independent activation domain (31). PPARs bind to DNA targets on peroxisome proliferator response elements as obligate heterodimers with the retinoid X receptors (RXRs) independent of their ligands (32). They also bind to other transcription factors to either repress or enhance their activity. Binding to RXR and DNA target sequences is of greater affinity and stability when the PPARs are bound to their ligands (33, 34). Most FAs can activate and act as ligands for PPARs but in general, PPARs have a preference for long-chain polyunsaturated fatty acyls (PUFAs) (35). Three different PPAR forms have been cloned. PPAR α and β/δ are associated with highly oxidative metabolically active tissues such as cardiac muscle, brown adipose tissue, and liver whereas PPARγ is more ubiquitously distributed. PPARα is considered a master regulator of lipid catabolic processes and increases transcription of genes associated with lipid catabolism. PPARβ/δ increases metabolism of LCFAs in muscle and decreases glycolaysis during sustained exercise (36).

Peroxisome proliferator-activated receptor γ acts as a nutrient sensor for non-esterified LCFAs and alters transcription in cells to promote their storage as triacyl glycerides (37). PPARγ activates genes associated with transport of FAs across the plasma membrane (CD36 and FABP4). It also activates genes associated with storage of FAs as TGs such as the perilipins. PPARγ also controls metabolic shift from glucose oxidation to TG production by inhibition of pyruvate dehydrogenase and upregulation of enzymes involved in triose production necessary for FA esterification (38, 39).

All three of the PPAR family members have been shown to play a role in T cell activation, proliferation, and differentiation into Th1, Th2, Th17, and Treg lineages (40) PPARγ is thought to inhibit the activity of nuclear factor of activated T cells and subsequent interleukin (IL)-2 production by T cells (40–42). PPARγ agonists have also been shown to potently inhibit the induction of inflammation in *in vivo* colitis models (43, 44). The decision of CD4 T cells to differentiate into Th17 or Treg is governed, in part, by PPARγ activity. Conversion of naïve effector T cells into TGFβ-induced Treg (iTreg) induction is enhanced in the presence of the PPARγ ligand ciglitazone (45). Conversely PPARγ deficiency in T cells results in elevated disease scores in the mouse model for multiple sclerosis, experimental autoimmune encephalomyelitis (EAE), with greater numbers of central nervous system (CNS) infiltrating Th17 cells (46). PPARα and PPAR β/δ also have a potent anti-inflammatory role in EAE models. Treatment with gemfibrozil, a PPARα agonist inhibited EAE disease severity *via* skewing of T cells to a Th2 phenotype (47). Agonists of PPARβ/δ GW-0742 could also inhibit EAE severity partially *via* reduction of IL-1β production (48). PPARγ is upregulated in a specialized adipose tissue-resident Treg subset described in more detail below.

### Sterol Regulatory Element-Binding Proteins (SREBPs)

Sterol regulatory element-binding proteins are transcription factors, which activate all genes necessary for FA synthesis (49). They have a basic helix-loop-helix leucine zipper structure (23) and exist in two forms produced by differential exon usage, resulting in two separate promoters (50). SREPB1 activates genes involved in *de novo* lipogenesis whereas SREBP2 activates genes necessary for cholesterol synthesis and uptake. SREBPs are made as cytoplasmic precursor molecules and must be cleaved and transported to the nucleus before binding to their target genes (51). When there is sufficient cholesterol in the cellular environment, SREBPs complex with SREBP cleavage-activating protein (SCAP) a cholesterol sensor and chaperone (52, 53), which keeps the complex tethered to the endoplasmic reticulum in an inactive state (51). Reduction in the cholesterol concentration within the cell results in a conformational change in SCAP allowing SCAP/SREBP translocation to the Golgi apparatus. Here, SREBPs are sequentially cleaved by site 1 and site 2 proteases (S1P and S2P) releasing the N-terminal portion to transfer into the cell nucleus (54, 55). SREBPs bind to consensus regions in the promoters of their target genes termed insulin response elements. Target genes include those involved in synthesis of cholesterol from acetyl-CoA and transfer of cholesterol into the cell (49). They are controlled by phosphorylation and degraded by ubiquitination. SREBs have been shown to be crucial to licensing blastogenesis and expansion of CD8 T cells in response to viral infection (56). In this context, SREBs are required for supplying sufficient lipids for membrane synthesis to allow expansion. An overview of lipid fates within T cells is shown in Figure 2.

## A Role for LIPID Metabolism in T-Cell Subset Differentiation

### Metabolic Requirements Change during the Life of a T Cell

T cells change their metabolic mode to fulfill requirements placed upon them during development, activation, proliferation, and formation of memory. Activation signals *via* the T cell receptor induce a program of blast formation and extensive cell division. This is both energy-demanding and requires formation of new cellular components such as membranes, DNA, and proteins for increased cell size and mitosis. To meet these requirements activated T cells adapt to preferentially utilize glucose and aerobic glycolysis to fuel ATP production. Aerobic glycolysis is less efficient in production of ATP than oxidative phosphorylation (OXPHOS). Despite this T cells take advantage of the fact that products of the glycolytic, and linked pentose phosphate, and trichloroacetic acid pathways such as citrate and ribose-5-phosphate are precursors of membrane and nucleic acids, both required for organelle biogenesis during proliferation. Microenvironmental cues in the form of cytokines and co-stimulatory triggers guide T cells down different functional routes including multiple CD4 helper T cell subsets and regulatory T cells. In addition to these cues, it is becoming clear that metabolic substrate availability is also a driver of T cell fate, discussed below. At the culmination of an immune response, T cells either enter into apoptosis or revert to non-dividing memory T cells. Memory T cells revert to lipid oxidation to generate energy, being quiescent they are less dependent on organelle biogenesis.

### CD8 T Cells

CD8 T cells have been shown to have distinct requirements for FAs to fuel memory differentiation, and subset specialization (Figure 3). Pearce and colleagues (57) generated CD8 effector and central memory cells (T_CM_) *in vivo*, in a murine listeria monocytogenes-ovalbumin (OVA) infection model of OT-1 chicken egg OVA-specific TCR transgenic mice. T_CM_ have elevated fatty acid oxidation (FAO) when compared with effector CD8 T cells. Paradoxically, these cells also take up less FAs from their environment. Instead, CD8 T_CM_, in this model, use extracellular-derived glucose to fuel FA synthesis and TG synthesis. The cells then hydrolyze these lysosomally stored TGs using the enzyme lysosomal acid lipase in a process termed “cell-intrinsic lipolysis.” The reason why CD8 T_CM_ in this model engage this type of “futile cycle” is not currently understood.

Subset specialization in memory CD8 T cell metabolism has recently been reported (26). Kupper and colleagues demonstrated that tissue-resident memory CD8 T cells (T_RM_ cells) in human and mouse differ from T_CM_. T_RM_ are a tissue-resident population of memory T cells, which may be CD4+ or CD8+, which reside in barrier epithelia, and persist for long periods to protect the host from pathogenic bacteria and viruses (58–60). CD8 T_RM_ are transcriptionally distinct from central memory cells (61). When OT-1 TCR transgenic mice were inoculated with recombinant vaccinia virus expressing OVA, they showed that the T_RM_ that this protocol generated have elevated expression of proteins involved with FA uptake and FAO compared with T_CM_. This included expression of FABPs 4 and 5 (FABP4,5) (Figure 3). T_RM_ have an increased requirement for FA metabolism compared with central memory cells or effector CD8 T cells. T_RM_ lacking FABP4 and 5, or those treated with inhibitors of FAO have attenuated function and reduced persistence in skin epithelia (26).

Thus, both subset specialization and environmental localization have a role in determining the metabolic requirements of CD8 memory T cells, with central memory cells appearing to be less dependent on environmental FAs than their tissue-resident counterparts for stability and functional competence.

### CD4 T Cells

A fundamental question that remains to be answered in immunometabolism is whether environmental metabolic substrate availability drives T cell differentiation, or whether cell-intrinsic programs dictate metabolic requirements which are then selected by the environment. Several recent publications have provided evidence that CD4 cell fate determination is likely the combination of both processes. The choice of development into either iTreg or Th17 lineage is determined by cell extrinsic and intrinsic cues. These include cytokines TGFβ, IL-6, IL-23, metabolic substrate availability, transcription factor expression [Foxp3, RAR-related orphan receptor gamma t (RORγt)], and the activity of key metabolic enzymes (12, 26, 40–42, 46, 57, 62–69). CD4 Teff and iTreg have been reported to have different metabolic requirements (70). Rathmell and colleagues reported that Teff have elevated glucose transporter 1 expression, preferential requirement for glucose, and higher levels of glycolysis than iTreg (70, 71), which rely on FAs as their preferred metabolic substrates. In the absence of FAs or inhibited FAO, iTreg are unable to develop *in vitro*. iTreg have elevated AMP-activated protein kinase (AMPK) compared with effector T cells, and activation of AMPK is sufficient to skew differentiation toward the iTreg lineage both *in vitro* and *in vivo* (70). In this study, palmitate exposure induced apoptosis selectively in Teff, suggesting that availability of lipids may select for preexisting Treg in addition to imparting a metabolic advantage. Indeed, Foxp3 expression is sufficient to re-program cells to upregulate many proteins and enzymes associated with FAO and mitochondrial OXPHOS (65) including many components of the mitochondrial electron transport system. Expression of Foxp3 imparts selective survival of cells exposed to saturated LCFAs palmitate and stearate at moderately raised physiological concentrations. This effect is dependent on FAO in these cells as inhibitory drugs, targeting several enzymes of the FA β-oxidation pathway, reverse the protective effect (65).

Foxp3 may be required to protect Treg in environments high in FAs, but also in environments low in glucose and high in lactate, such as the intestinal tract and ischemic tissues (72). Foxp3 was reported to suppress Myc and glycolysis, thus enhancing OXPHOS and NAD regeneration, protecting Treg from lactate-mediated inhibition of proliferation (62). While favoring immune tolerance in ischemic tissues and the gut, these mechanisms may also be detrimental to immune defense against tumors.

There are several reported metabolic checkpoints involved in controlling whether CD4 T cells develop into Th17 effector cells or iTreg under identical environmental conditions. A recent report highlighted the role of the enzyme pyruvate dehydrogenase kinase (PDHK) in selective regulation of T cell differentiation and inflammation (64). Pyruvate dehydrogenase was identified as a bifurcation point in the choice between glycolytic and oxidative metabolism (Figure 2). Th17 cells express higher levels of PDHK than Th1 or Treg, and inhibition of PDHK resulted in preferential expansion of Treg. This effect was partly due to the effects of elevated reactive oxygen species generated following PDHK inhibition, to which Treg are resistant.

A selective requirement for *de novo* FA synthesis has been reported for Th17 cell development and functions (63). Th17 cells are thought to favor a glycolytic/lipogenic mode of metabolism for their development which requires acetyl-CoA-carboxylase 1 (ACC1). ACCs catabolize the ATP-dependent carboxylation of acetyl-CoA to malonyl-CoA, essential for FA synthesis in the cytosol. Th17 cells use this pathway for production of cellular membrane phospholipids, whereas Treg preferentially take up exogenous FAs for this function. Inhibition of ACC1 in human and mouse T cells impairs the development of Th17 cells and preferentially allows development of Treg (63).

Fatty acid metabolism has also been reported to control pathogenicity within the Th17 compartment (68). Activation of T cells in the presence of the cytokines TGFβ and IL-6 promotes differentiation of IL-17-producing cells, which are poor at inducing EAE. Addition of IL-23 to these cultures induces cells which produce IL-17 and are also potent inducers of EAE pathology. These cells are termed “non-pathogenic” and “pathogenic,” respectively. Kuchroo and colleagues identified CD5 molecule like/apoptosis inhibitor expressed by macrophages as a molecule expressed in non-pathogenic but not pathogenic Th17 cells (Figure 3). CD5L modulates the intracellular lipidome through modifying FA synthesis *via* binding to FA synthase. In this way, it inhibits FA synthesis. CD5L also alters the FA composition including the inhibiting the amount of PUFAs such that cholesterol biosynthesis is inhibited through inhibition of the enzymes sc4mol and cyp51 (Figure 3) (68). Consequently, the concentration of available RORγt ligands is reduced in the cell. They showed that saturated fatty acid (SFA) increased whereas PUFA decreased binding of RORγt to the *Il17* and *Il23r* loci. CD5L is a general inhibitor of Th17 pathogenicity as its removal converts non-pathogenic Th17 cells into pathogenic cells capable of causing inflammation *in vivo* (68).

### Cholesterol Biosynthetic Intermediates and T-Cell Functions

**Figure 4:**
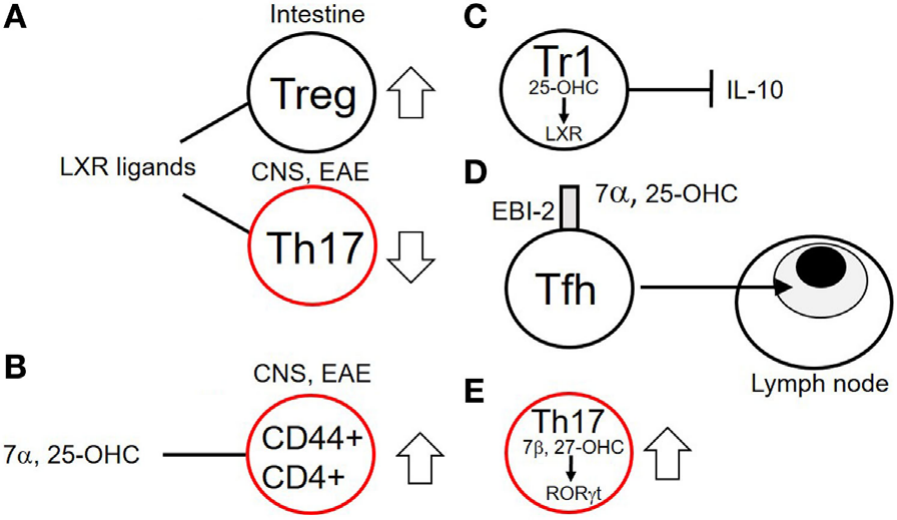
Effects of cholesterol biosynthetic intermediates on T cell functions. **(A)** Liver X receptor family (LXR) ligands increase Treg numbers in the intestine while decreasing numbers of Th17 cells in the central nervous system (CNS) of mice with experimental autoimmune encephalomyelitis (EAE). **(B)** The oxysterol 7α,25-OHC increases numbers of activated CD44+ CD4+ T cells in the CNS of mice with EAE. **(C)** Tr1 cells produce the sterol 25-OHC which *via* binding to the LXR inhibits production of interleukin (IL)-10 in a negative feedback loop. **(D)** 7α,25-OHC *via* its plasma-membrane receptor EBI-2 promotes migration of T follicular helper (Tfh) cells to the T cell zones proximal to B cell follicles. **(E)** Th17 cells produce 7β,27-OHC, an endogenous ligand for the RAR-related orphan receptor gamma t (RORγt) transcription factor, necessary for their function. Cells outlined in red indicate potentially inflammatory subsets.

Cholesterol and its biosynthetic intermediates have profound effects on multiple aspects of immunity. These include roles in B lymphocyte homing to lymph nodes (73), control of viral replication (74), macrophage phagocytosis (75), inflammasome activation (76), antitumor responses of CD8 T cells (77), and neutrophil traps (78). Cholesterol metabolites, particularly oxysterols are increasingly being shown to have roles in T cell development, function, and migration (Figure 4) (75). Cholesterol derivatives signal in T cells *via* the liver X receptor family (LXR) of transcription factors. LXRα and LXRβ transcription factors have multiple positive and negative effects on transcription in many cell types (75). LXRα is predominantly expressed in adipose tissues where it controls genes involved in catabolism of cholesterol while LXRβ is expressed ubiquitously including in lymphocytes. The ligands for LXRs *in vivo* include cholesterol precursors and oxysterols. These include desmosterol, 24S-hydroxycholesterol, 25-hydroxycholesterol, and 27-hydroxycholesterol (79). LXRs not only control genes involved in cholesterol and FA biosynthesis but also suppress the activity of genes under control of NF-κB and AP-1 (80, 81). LXR ligation may have pro- or anti-inflammatory roles depending on the cell type and sterol involved. In EAE, LXR ligation is protective (Figure 4B) (82). Mice deficient in LXR have increased infiltration of inflammatory cells into the spinal cord and more severe demyelination (83). LXR ligand treatment of the EAE model results in decreased disease severity, decreased Th17 polarization, and a reduction of IL-17 (84). Pharmacological LXR agonists have been reported to enhance Treg differentiation, increasing the number of gut Treg in mice (Figure 4A) (85). Certain oxysterols have also been reported to play pro-inflammatory roles in EAE (86). Chalmin et al. reported a pro-inflammatory role for 7α, 24-hydroxy cholesterol (7α, 25-OHC) in EAE (86). They showed that 7α, 25-OHC promoted increased migration into the CNS of activated CD44+ CD4+ T cells, *via* the cell-surface reporter EBI-2 (GPR183). Deletion of the enzyme responsible for production of 7α, 25-OHC, cholesterol 25 hydroxylase, reduced the severity of EAE by limiting trafficking of pathogenic CD44+ CD4+ cells into the CNS. A potentially pro-inflammatory role for 25-OHC has been described in IL-27-induced type-1 regulatory (Tr1) cells (Figure 4C) (87). These cells express 25-OHC and cholesterol 25 hydroxylase. 25-OHC inhibits IL-10 production *via* the LXR, inhibiting the regulatory potential of these cells. Cyster and colleagues described a mechanism for 7α, 25-OHC in mediating the correct migration of T follicular helper (Tfh) cells in the lymph node between the T cell zone and B cell follicle, *via* the receptor EBI-2 (Figure 4D) (88). A positive role for oxysterols has been established for Th17 function (Figure 4E) (89, 90). The transcription factor RORγt is expressed in lymphoid tissues and is crucial for development of thymocytes, lymph nodes, gut associated lymphoid tissue and Th17 cells (91–94). For optimal activity RORγt needs to bind to cholesterol derivatives *via* its ligand-binding domain. The oxysterols 7β, 27-dihydroxycholesterol (7β, 27-OHC) is the most potent oxysterol ligand for RORγt (89). Binding of 7β, 27-OHC enhances Th17 differentiation. Th17 endogenously produce both 7β,27-OHC and 7α,27-OHC, and it has been shown that mice lacking the enzyme responsible for production of 7β,27-OHC (CYP27A1) have a deficiency in Th17 cells (89). Inhibition of cholesterol esterification in CD8 T cells by inhibition of the enzyme ACAT1 has been shown to potentiate antiviral CD8 T cell functions (77). This was shown to be a result of elevated cholesterol in the plasma membrane, which enhances TCR signaling and formation of the immunological synapse. This result suggests that cholesterol metabolism may represent a novel target for cancer therapy.

### SCFAs and Colonic cTreg

Fatty acids have a major role in shaping a population of regulatory T cells resident in the mucosal layer of the colon (1, 3–5). cTreg play an important role in maintenance of tolerance to antigens derived from food and bacterial flora. These cells depend on resident gut bacteria of the *Bacteroides* and *Clostridia* species for their induction and function (2, 66). Gut bacteria are required to break down indigestible dietary fiber and carbohydrates. SCFAs such as acetate, propionate, and butyrate are produced by bacteria as a result of fermentation of such dietary components. The gut lumen has between 50 and 100 mM SCFA content (95); however, the concentration of SCFA in the gut lumen of germ free mice is markedly reduced compared with mice housed in specific pathogen-free conditions (4). The size of the gut cTreg pool is thought to be controlled by the bacterially derived SCFA concentration. Addition of propionate to the drinking water of germ-free mice increases their cTreg numbers (4) but has no effect on numbers of splenic, mesenteric, or thymic Treg numbers. Conversely, inhibition of colonic bacterial numbers with vancomycin results in a reduction in cTreg numbers, which is reversible by addition of SCFA to the drinking water of mice. The SCFA butyrate is an inhibitor of class I and IIa histone, deacetylases and, as such, has a potent effect on histone 3 acetylation surrounding the promoter and conserved non-coding regions 1 and 3 of the Foxp3 locus, regions essential for induction of peripheral Treg (1). SCFA induction of cTreg depends on the Foxp3 enhancer conserved non-coding sequence-1 (CNS1), showing that this induction is *via de novo* induction of Treg locally, as thymic Treg do not require CNS1 for their development (69). Maintenance of immune tolerance *via* SCFA Treg induction requires the SCFA receptors GPR43 on colonic epithelial cells in addition to GPR109A on dendritic cells and, in addition to enhancing Treg numbers, increases the tolerogenic properties of CD103-expressing colonic dendritic cells (5).

### Visceral Adipose Tissue Treg

Adaptations to preferential FA metabolism are seen in a specialized subset of Treg residing in lean (visceral) fat tissue, termed visceral adipose tissue Treg or VAT Treg (96–98). These cells accumulate in visceral fat early in life (98) and expand in an MHC/peptide and IL-33-dependent fashion (98). The cells differ from conventional Treg in several ways. They constitute a very high proportion of CD4+ T cells in adipose tissue (40–80%) (97) and have a transcriptional profile that is different from conventional Treg, overexpressing chemokine receptors CCR1 and CCR2 and IL-10 (97). They also overexpress several transcripts associated with FA metabolism such as diacylglycerol acyl transferase 1, CD36, and low-density lipoprotein receptor. These cells have a distinct T cell receptor repertoire from conventional Treg. Many of these differences are due to expression of the adipocyte master regulator transcription factor PPARγ which, together with Foxp3, cooperates to program their specialized function (96). PPARγ is necessary for VAT Treg to accumulate in visceral fat and to inhibit inflammation within obese fat, restoring responsiveness to insulin. Treg-specific knock ut of PPARγ results in fewer VAT Treg, but no change in splenic Treg number or function. Pioglitazone, a synthetic PPARγ agonist, increases the number of VAT Treg in high fat diet-fed obese mice but has no effect on the numbers of splenic Treg (98).

### FAs and Lipotoxicity

The increasing prevalence of obesity worldwide is leading to an epidemic of related health problems including diabetes and coronary artery disease. Much of the harm done to individuals with elevated body mass indices arises from raised plasma free fatty acid levels. This has been shown to trigger the metabolic syndrome (99). Adipocytes are adapted to store excess TGs as fat droplets, but non adipose cells such as pancreatic beta cells, hepatocytes and lymphocytes have a limited capacity to convert FFAs to TGs in fat droplets. In such cells exposure to elevated FFAs can result in cellular damage and ultimately cell death, a process called lipotoxicity (65, 100, 101).

Exposure of T cells to FAs and lipids in culture has varied effects depending on the type of FA and the concentration. Exposure of human Treg to high-density lipoproteins (HDL), but not LDLs significantly reduces these cells’ apoptosis in response to serum starvation in *in vitro* cultures, but has little protective effect on naïve and memory CD4 T cell survival under the same conditions (102). This was reported to be due to HDL operating *via* the scavenger receptor class B type I, increasing spare respiratory capacity and basal respiration in Treg. Low doses of FAs may induce T cell activation with higher doses resulting in apoptosis (103–106). Moderately raised physiological levels of saturated FAs induce in primary T cells or T cell lines cytochrome-*c* release from mitochondria, loss of mitochondrial membrane potential, externalization of phosphatidyl serine (65), caspase activation, and DNA fragmentation (105) indicating an apoptotic mechanism. Loss of CD4 T cells in non-alcoholic fatty liver disease has been attributed to mitochondrial damage and apoptosis induced by reactive oxygen species released in response to linoleic acid (107).

T cells can convert excess exogenous FAs into neutral lipids such as triacyl glycerides and cholesterol esters (Figure 2). Channeling of dietary LCFAs to distinct metabolic routes has been shown to correlate with their propensity to induce lipotoxicity in many non-lymphoid cell types (100). This channeling of FAs into neutral lipids, stored as intracellular lipid droplets is protective to the cell. In general saturated LCFAs can induce cytotoxicity whereas monounsaturated FAs are non-toxic or are cytoprotective to cells (101). It has been shown in multiple cell types that addition of monounsaturated fatty acid (MUFA) to cells dose dependently protects against the cytotoxic effects of SFA by inhibiting FA synthesis and channeling FAs to TGs (100, 108–111). The protective effect of MUFAs in lipotoxicty occurs *via* a mechanism involving the endoplasmic reticulum MUFA sensor UBXD8. UBXD8 inhibits TG synthesis by blocking conversion of diacylglycerides to TGs. An excess of MUFAs relieves this inhibition, licensing production of inert TGs, and thus protecting the cell from lipotoxicity (108). It remains to be seen whether this mechanism of protection from lipotoxicity by MUFAs operates in T cell subsets; however, upregulation of FAO pathways by Foxp3 endows Treg with a selective survival advantage during exposure to raised SFA concentrations *in vitro* (65).

### Potential Therapeutic Applications

Because the metabolism of T lymphocytes is so closely linked to their activation, differentiation, and survival, there is tremendous interest in manipulating metabolic processes for therapeutic purposes. This topic has been well reviewed recently (112–115) so only a brief summary of potential lipid metabolic drug targets will be described here. It is likely that many existing drugs used to normalize metabolic imbalances might be “repositioned” for use in other indications including treatment of autoimmunity and graft-versus-host disease (GVHD). Statins are drugs that inhibit cholesterol synthesis by inhibiting the action of the enzyme HMG-CoA-reductase, an enzyme that generates mevalonate, a key intermediate in this pathway. Prescribed for the treatment of raised plasma cholesterol, they are one of the most prescribed drugs in the world. Statins have a potent inhibitory effect on differentiation of Th17 cells, skewing differentiation toward Treg (116, 117). Simvastatin was shown to promote Treg differentiation and inhibit Th17 development under Th17 polarization conditions. These effects were dependent on inhibition of protein geranylgeranylation by the drug (116). Simvastatin may also inhibit the inhibitory SMADS; SMAD6 and SMAD7, the consequence being that the drug synergizes with low amounts of TGFβ to generate pTreg (117). Statins also reduce the intracellular concentration of desmosterol, a cholesterol precursor and potent endogenous RORgt ligand (118). It is likely that this property of statins may be exploited for inhibition of Th17-mediated inflammatory conditions in the future.

The PPARα agonists, gemfibrozil, and fenofibrate are oral drugs widely prescribed for the treatment of hypertriglyceridemia. Both are able to treat ongoing signs of EAE in mice (119). Inhibition of PPARα in T cells was shown to skew the immune response, promoting IL-4 production and inhibiting IFN-γ. These results suggest that the PPARα agonist family of drugs might be repositioned for use in autoimmune diseases such as MS.

Activated inflammatory T cells in GVHD have been shown to rely on OXPHOS for proliferation (120). These alloreactive effector T cells have a strong preference for FAs to fuel this metabolic mode, upregulating transcriptional coactivators for lipid catabolism and increasing their FA uptake. Inhibition of FA beta-oxidation with etomoxir reduced the survival of alloreactive effector T cells but has no effect on syngenic T cell expansion. These observations raise the prospect of modulating lipid metabolism to selectively inhibit alloreactive T cells in GVHD using drugs such as etomoxir or perhexiline (121).

## Conclusion

T cell differentiation, functions, and survival are increasingly demonstrated to be linked to processes of metabolism, particularly lipid metabolism. CD4 and CD8 subset differentiation, memory, effector function, and survival are dependent on various aspects of lipid synthesis, catabolism, and storage. There is intense interest in revealing aspects of metabolism, which are uniquely required for particular T cell subsets, so as to identify opportunities for therapeutic manipulation. The challenges, as the field progresses, will be to identify those differences that are “programmed” by transcription factors as compared with those which result from environmental cues. The links between metabolic processes, cell signaling, genetic, and epigenetic control are just beginning to be identified and represent an exciting new dimension in the area of immune regulation.

## Author Contributions

DH wrote the manuscript. AB, AN, SC, and HW cowrote the manuscript.

## Conflict of Interest Statement

The authors declare that the research was conducted in the absence of any commercial or financial relationships that could be construed as a potential conflict of interest.

## Abbreviations

ACC1, acetyl-CoA carboxylase 1; AMPK, AMP-activated protein kinase; CD5L/AIM, CD5 molecule like/apoptosis inhibitor expressed by macrophages; CNS, central nervous system; CPT1, carnitine palmitate transferase; DAG, diacylglyceride; DCA, dichloro acetic acid; DGAT, diacylglycerol acyl transferase; EAE, experimental autoimmune encephalomyelitis; FA, fatty acid; FABP, fatty acid-binding protein; FAO, fatty acid oxidation; FATP, fatty acid transport protein; FFA, free fatty acid; GPR, G protein-coupled receptor; HDL, high-density lipoprotein; IL, interleukin; LAL, lysosomal acid lipase; GLUT1, glucose transporter 1; LCFA, long-chain fatty acid; LDL, low-density lipoprotein; LDLR, low-density lipoprotein receptor; MCFA, medium-chain fatty acid; MUFA, monounsaturated fatty acid; NFAT, nuclear factor of activated T cells; OXPHOS, oxidative phosphorylation; PDHK, pyruvate dehydrogenase kinase; SCAP, SREBP cleavage-activating protein; PPAR, peroxisome proliferator-activated receptor; RORγt, RAR-related orphan receptor gamma t; RXR, retinoid X receptor; SCFA, short-chain fatty acid; SFA, saturated fatty acid; SNP, single-nucleotide polymorphism; SREBP, sterol regulatory element-binding protein; TCA, trichloroacetic acid; TG, triglyceride; VLCFA, very long-chain fatty acid; VLDL, very low-density lipoprotein.
